# Effect of Intranasal Dexmedetomidine or Midazolam for Premedication on the Occurrence of Respiratory Adverse Events in Children Undergoing Tonsillectomy and Adenoidectomy: A Randomized Clinical Trial

**DOI:** 10.1001/jamanetworkopen.2022.25473

**Published:** 2022-08-09

**Authors:** Fangming Shen, Qin Zhang, Yahui Xu, Xinghe Wang, Jiayi Xia, Chao Chen, He Liu, Yueying Zhang

**Affiliations:** 1 Jiangsu Province Key Laboratory of Anesthesiology, Xuzhou Medical University, Xuzhou, Jiangsu, China; 2 Department of Anesthesiology, The Affiliated Hospital of Xuzhou Medical University, Xuzhou, Jiangsu, China; 3 The Children’s Hospital of Xuzhou Medical University, Xuzhou, Jiangsu, China; 4 Department of Anesthesiology, Huzhou Central Hospital, The Affiliated Huzhou Hospital, Zhejiang University School of Medicine, Huzhou, Zhejiang, China

## Abstract

**Importance:**

Perioperative respiratory adverse events (PRAEs) are the most common complication during pediatric anesthesia, and they may be affected by the administration of preoperative sedatives.

**Objective:**

To investigate the effect of intranasal dexmedetomidine or midazolam used for premedication on the occurrence of PRAEs.

**Design, Setting, and Participants:**

This single-center, double-blind, randomized clinical trial was conducted among children aged 0 to 12 years undergoing elective tonsillectomy and adenoidectomy from October 2020 to June 2021 at Children’s Hospital of Xuzhou Medical University, Xuzhou, China. Data analysis was performed from June to October 2021.

**Interventions:**

Children were randomly assigned to 3 groups: the midazolam group received intranasal midazolam (0.1 mg/kg), and the dexmedetomidine group received intranasal dexmedetomidine (2.0 μg/kg) for premedication. The normal saline group received intranasal 0.9% saline for control.

**Main Outcomes and Measures:**

The primary outcome was the difference in the incidence of PRAEs among the 3 groups. The secondary outcomes were the frequency of the individual PRAEs, including the incidence of such events during the induction and recovery periods, postoperative emergence delirium, postoperative pain score, sedation success rate, and heart rate values.

**Results:**

A total of 384 children (median [IQR] age, 7 [5-10] years; 227 boys [59.1%]) were enrolled and randomized; 373 data sets were available for intention-to-treat analysis (124 children in the midazolam group, 124 children in the dexmedetomidine group, and 125 children in the normal saline group). After the data were adjusted for age, sex, American Society of Anesthesiologists physical status, body mass index, obstructive sleep apnea, upper respiratory tract infection, and passive smoking, children in the midazolam group were more likely to experience PRAEs than those in the normal saline group (70 of 124 children [56.5%] vs 51 of 125 children [40.8%]; adjusted odds ratio [aOR], 1.99; 95% CI, 1.18-3.35), whereas the dexmedetomidine group had a significantly lower PRAEs incidence than the normal saline group (30 of 124 children [24.2%] vs 51 of 125 children [40.8%]; aOR, 0.45; 95% CI, 0.26-0.78). Compared with the dexmedetomidine group, the midazolam group had a higher risk of PRAEs (aOR, 4.44; 95% CI, 2.54-7.76), but no other serious clinical adverse events were observed.

**Conclusions and Relevance:**

In this randomized clinical trial, intranasal midazolam used for premedication was associated with increased incidence of PRAEs, whereas premedication with intranasal dexmedetomidine was associated with reduced incidence of PRAEs. Where clinically appropriate, anesthesiologists should consider using intranasal dexmedetomidine for sedation in children undergoing tonsillectomy and adenoidectomy.

**Trial Registration:**

Chinese Clinical Trial Register Identifier: ChiCTR2000038359

## Key Points

**Question:**

Do preoperative sedatives such as midazolam and dexmedetomidine have a protective effect against perioperative respiratory adverse events (PRAEs) while achieving adequate sedative effect?

**Findings:**

In this randomized clinical trial that included 384 children undergoing tonsillectomy and adenoidectomy, the incidence of PRAEs was 24.2% in the dexmedetomidine (2.0 μg/kg) group, 56.5% in the midazolam (0.1 mg/kg) group, and 40.8% in the normal saline group.

**Meaning:**

Intranasal midazolam used for premedication was associated with increased incidence of PRAEs, whereas premedication with dexmedetomidine was associated with reduced incidence of PRAEs.

## Teaser

This randomized clinical trial investigates the effect of intranasal dexmedetomidine or midazolam used for preoperative sedation on the occurrence of perioperative respiratory adverse events (PRAEs) among children undergoing tonsillectomy and adenoidectomy.

## Introduction

Perioperative respiratory adverse events (PRAEs) are the most common complication during pediatric anesthesia,^1,2^ manifested as minor adverse events (oxygen desaturation, airway obstruction, coughing, or wheezing) and major adverse events (laryngospasm and bronchospasm). The occurrence of these complications can prolong hospitalization time, increase hospitalization costs, and bring varying degrees of physical and psychological trauma to children and parents.^3,4^ A substantial proportion of children undergoing tonsillectomies experience PRAEs, with a prevalence of up to 50%.^5,6^ Independent risk factors include age 6 years and younger, upper respiratory tract infection (URTI), lung disease, obesity, obstructive sleep apnea (OSA), and passive smoking.^7^ These factors are very common in children undergoing tonsillectomy and adenoidectomy.

Clinicians have explored various strategies to minimize PRAEs, including but not limited to the use of laryngeal mask airways, intravenous induction of anesthesia (vs mask induction), and lidocaine topicalization of the airway.^8,9,10^ However, preoperative strategies are needed to provide anesthesiologists a comprehensive approach for high-risk children.

Pediatric patients can experience substantial anxiety and distress during the perioperative period. The use of sedative premedication may help to reduce their anxiety and minimize the emotional trauma, but there are no preferred recommendations or well-documented clinical studies to guide our choice of a certain sedative to decrease the incidence of PRAEs. Midazolam and dexmedetomidine, which are the most common preoperative sedatives used for children, have been commonly used in recent years,^11,12,13^ but their effect on PRAEs is still unclear.

There are contradictory studies of midazolam and PRAEs: previous studies^14,15^ showed that preoperative midazolam seemed to increase the incidence of PRAEs, but a multicenter trial^16^ showed that midazolam had a preventive effect on PRAEs. Dexmedetomidine has been proven to be effective in reducing the occurrence of PRAEs in children with congenital heart disease,^17^ but there is no evidence to support this protective effect in the general population undergoing tonsillectomy and adenoidectomy. However, those previous studies are mostly observational, and, to our knowledge, there are still no randomized clinical trials with high-quality evidence to study the effect of the preoperative sedatives midazolam and dexmedetomidine on PRAEs.

To address this inconsistency and the small samples included in previous studies, we conducted a prospective, single-center, double-blind, randomized clinical trial to investigate the effect of intranasal dexmedetomidine or midazolam on the occurrence of PRAEs in children undergoing tonsillectomy and adenoidectomy. We hypothesized that preoperative sedatives, intranasal dexmedetomidine or midazolam, would reduce the occurrence of PRAEs.

## Methods

### Study Design and Population

This trial was performed at the Children’s Hospital of Xuzhou Medical University, Xuzhou, China, from October 1, 2020, to June 30, 2021. The study protocol was approved by the Medical Ethics Committee of the Children’s Hospital of Xuzhou Medical University and was registered in the Chinese Clinical Trial Registration Center on September 21, 2020. This report follows the Consolidated Standards of Reporting Trials (CONSORT) reporting guideline for randomized studies.^18^ The full trial protocol is available in Supplement 1.

Our preliminary data suggested an approximate incidence of PRAEs in the midazolam, dexmedetomidine, and normal saline groups of 60%, 20%, and 40%, respectively. A difference between groups that reached 20% was considered clinically meaningful. After adjusting for multiplicity from making 3 pairwise comparisons, a sample size of 115 per group at a *P* < .017 two-sided significance level provided an 80% power to detect a 20% difference in the rate of PRAEs among the 3 groups using the χ^2^ test. Allowing for 10% loss of cases because of unusable or missing data, we aimed to recruit 128 participants in each group, or 384 cases in total.

The CONSORT flowchart is shown in the Figure. Children aged 0 to 12 years with American Society of Anesthesiologists physical status categories I and II were eligible for inclusion if they were undergoing elective tonsillectomy with or without adenoidectomy. The exclusion criteria were (1) known cardiopulmonary diseases (eg, uncorrected congenital heart disease, primary or secondary pulmonary hypertension, tumors, or structural lung diseases); (2) neuromuscular diseases; (3) body mass index (calculated as weight in kilograms divided by height in meters squared) greater than 30; (4) severe URTI and the anesthesiologist recommended delaying surgery; (5) allergy to either midazolam or dexmedetomidine; and (6) parents refusing to allow their children to participate.

**Figure.**
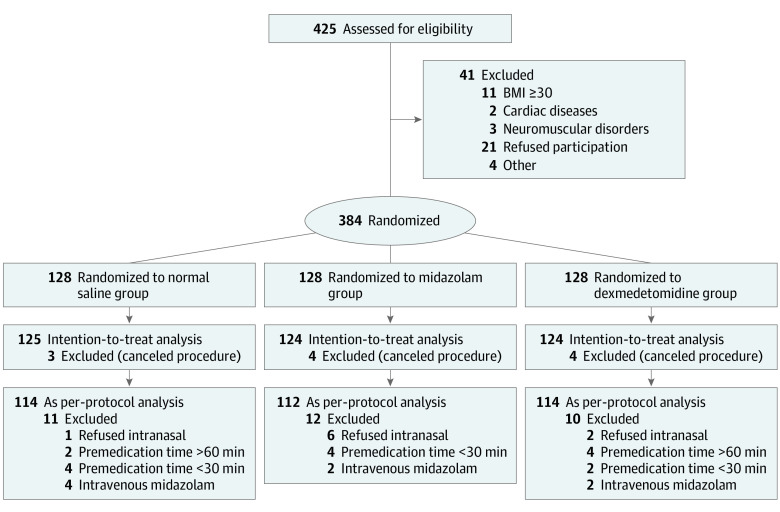
CONSORT Diagram BMI indicates body mass index (calculated as weight in kilograms divided by height in meters squared).

### Study Procedures and Interventions

On the day before the operation, potential participants were identified from the elective surgery list by a member of the research team, baseline data and risk factors were collected (eAppendix 1 in Supplement 2), and written informed consent was obtained from the children’s parents or guardians. All children were routinely required to preoperatively fast 8 hours for solids and 2 hours for clear liquids. Upon arrival in the anesthetic preparation room, children received intranasal premedication at approximately 30 to 60 minutes. Group allocations were kept in opaque sealed envelopes sequentially numbered and disclosed by a health care practitioner not directly involved in the children’ clinical management and data collection. Each code according to a random number was used to divide the children into 3 groups: children received intranasal midazolam (0.1 mg/kg) or dexmedetomidine (2.0 μg/kg), with 0.9% saline added to make a final volume of 1 mL; the control group was given 1 mL of 0.9% saline. The prepared drug solution was administered cautiously in both nostrils using a needleless 1-mL syringe, drop by drop, and the children were positioned on the parent’s lap in a recumbent position during administration. Midazolam took effect after approximately 10 to 15 minutes but could also achieve a satisfactory sedative effect after 30 minutes.

The anesthesia induction (inhalational or intravenous induction) was determined by the responsible anesthetist independently. Preoxygenation was routinely used. Tracheal tubes for airway management were used in all children. After the end of surgery, secretions and intraoperative irrigation fluid in the mouth were sucked out to avoid aspiration, and then children with a tracheal tube were transferred to the postanesthesia care unit (PACU). Tracheal extubation was undertaken when the child was awake by a specialized pediatric anesthesiologist in the PACU. Neostigmine was used if the child presented with residual muscle relaxation. After extubation, postoperative pain was assessed using the Wong-Baker Pain Scale (eAppendix 1 in Supplement 2), and if the score was more than 4, fentanyl (0.5-1.0 μg/kg) was administered for pain management. Patients were returned to the ward when their Steward score was greater than 4 (eAppendix 1 in Supplement 2). The anesthetic management was performed according to Chinese anesthesiology guidelines and expert consensus, and there was no deviation. More details about the protocol are shown in Supplement 1.

### Outcome Measure

The primary outcome was the difference in the incidence of PRAEs among the 3 groups. The secondary outcomes were the frequency of the individual PRAEs, the incidence of PRAEs during the induction and recovery periods (the time from the end of surgery to discharge from PACU), postoperative pain score, sedation success rate (Funk score^19^), postoperative emergence delirium, and heart rate values (eAppendix 1 in Supplement 2).

### Randomization and Masking

The children were allocated to the 3 study groups using computer-generated randomization, with group allocation and study number concealed in sealed envelopes. The intranasal drugs were prepared in a 1-mL syringe by an anesthesia nurse who was not involved in the study. The active drug or placebo was administered by a fully trained anesthesiologist. All researchers directly involved in the study were blinded to the drug being administered.

### Statistical Analysis

 Data analysis was performed from June to October 2021. Data were analyzed using SPSS statistical software version 26.0 (IBM). The Kolmogorov-Smirnov test was used to determine whether the continuous data conformed to the normal distribution. The quantitative variables that obey normal distribution are presented as mean (SD), and nonnormally distributed data are represented by median and IQR. Binomial variables are expressed as rate. The continuous data of normal distribution were analyzed by 1-way analysis of variance, and the continuous data of nonnormal distribution among the 3 groups were analyzed by the Kruskal-Wallis rank-sum test. Categorical data were analyzed using the χ^2^ test, and the *P* value was adjusted according to Bonferroni method and fixed at .017 for pairwise comparison. *P* < .05 was considered to indicate significance.

Outcome analyses were performed in the intention-to-treat population, and a per-protocol analysis was also performed for the primary end point. The primary outcome was analyzed using χ^2^ test or Fisher exact test, and the crude odds ratio (OR) and 95% CI were calculated. The adjusted OR (aOR) and 95% CI were calculated for both primary and secondary outcomes. Age, sex, American Society of Anesthesiologists physical status, body mass index, URTI, passive smoking, and OSA were adjusted for using binary logistic regression.

## Results

### Patients' Characteristics and Operative Data

A total of 384 children (median [IQR] age, 7 [5-10] years; 227 boys [59.1%]) were recruited (Figure). Of these, 11 participants were excluded from analysis owing to canceled procedures. Complete data sets were available from the remaining 373 participants (124 in the midazolam group, 124 in the dexmedetomidine group, and 125 in the normal saline group) and were evaluated for the intention-to-treat analysis; 340 cases were evaluated for the per-protocol analysis. Table 1 provides a detailed overview of participant demographics and general clinical history, which were similar among groups.

**Table 1.** Characteristics of the Patients at Baseline

Characteristic	Patients, No. (%) (N = 373)		
	Normal saline (n = 125)	Midazolam (n = 124)	Dexmedetomidine (n = 124)
Sex			
Female	53 (42.4)	49 (39.5)	50 (40.3)
Male	72 (57.6)	75 (60.5)	74 (59.7)
Age group, y			
0-3	12 (9.6)	17 (13.7)	16 (12.9)
4-6	33 (26.4)	37 (29.8)	38 (30.6)
7-9	40 (32.0)	38 (30.6)	40 (32.3)
10-12	40 (32.0)	32 (25.8)	30 (24.2)
Body mass index, median (IQR)^a^	17.2 (15.4-19.1)	15.9 (14.6-18.3)	16.3 (14.6-18.4)
American Society of Anesthesiologists physical status category			
I	36 (28.8)	49 (39.5)	50 (40.3)
II	89 (71.2)	75 (60.5)	74 (59.7)
Upper respiratory tract infection	43 (34.4)	45 (36.3)	35 (28.2)
Asthma	2 (1.6)	0	0
Allergy	16 (12.8)	16 (12.9)	20 (16.1)
Past or present eczema	13 (10.4)	17 (13.7)	25 (20.2)
Passive smoking	55 (44.0)	49 (39.5)	57 (46.0)
Obstructive sleep apnea	95 (76.0)	103 (83.1)	96 (77.4)
Preterm delivery	13 (10.4)	9 (7.3)	11 (8.9)
Time from premedication to induction, median (IQR), min	30.0 (30.0-35.0)	30.0 (30.0-30.0)	30.0 (30.0-33.7)
Induction of anesthesia			
Intravenous	109 (87.2)	113 (91.1)	108 (87.1)
Inhalation	16 (12.8)	11 (8.9)	16 (12.9)
Type of surgery			
Tonsillectomy	4 (3.2)	2 (1.6)	1 (0.8)
Adenoidectomy	25 (20.0)	21 (16.9)	28 (22.6)
Tonsillectomy plus adenoidectomy	96 (76.8)	101 (81.5)	95 (76.6)
Anesthesia duration, median (IQR), min	45.0 (35.0-60.0)	45.0 (35.0-63.8)	45.0 (35.0-55.0)
Surgery duration, median (IQR), min	40.0 (25.0-50.0)	40.0 (30.0-50.0)	35.0 (25.0-45.0)

^a^ Body mass index is calculated as weight in kilograms divided by height in meters squared.

### Primary Outcome

Table 2 shows the incidence of PRAEs among the 3 groups, using both the crude ORs and aORs. Children in the midazolam group were more likely to experience PRAEs than those in the normal saline group after adjusting for age, sex, American Society of Anesthesiologists status, body mass index, OSA, URTI, and passive smoking (70 of 124 children [56.5%] vs 51 of 125 children [40.8%]; aOR, 1.99; 95% CI, 1.18-3.35), whereas the dexmedetomidine group had a significantly lower PRAEs incidence than the normal saline group (30 of 124 children [24.2%] vs 51 of 125 children [40.8%]; aOR, 0.45; 95% CI, 0.26-0.78). Compared with the dexmedetomidine group, the midazolam group had a higher risk of PRAEs (aOR, 4.44; 95% CI, 2.54-7.76). eFigure 1 in Supplement 2 shows the comparison of incidence of PRAEs among the 3 groups. Results of the per-protocol analysis are shown in eTable 1 in Supplement 2.

**Table 2.** Comparison of the Incidence of Each Individual PRAE Among the 3 Groups Over the Perioperative Period (From Induction of Anesthesia to Discharge From the Postanesthesia Care Unit) for Intention-to-Treat Analysis

PRAEs	Patients, No. (%)			aOR (95%CI)		
	Normal saline (n = 125)	Midazolam (n = 124)	Dexmedetomidine (n = 124)	Midazolam vs normal saline	Dexmedetomidine vs normal saline	Midazolam vs dexmedetomidine
Any unadjusted	51 (40.8)	70 (56.5)	30 (24.2)	1.88 (1.14-3.11)^a^^,^^b^	0.46 (0.27-0.80)^a^^,^^b^	4.06 (2.36-6.99)^a^^,^^b^
Any adjusted^c^	51 (40.8)	70 (56.5)	30 (24.2)	1.99 (1.18-3.35)^a^	0.45 (0.26-0.78)^a^	4.44 (2.54-7.76)^a^
Major	3 (2.4)	13 (10.5)	3 (2.4)	4.29 (1.17-15.75)	0.83 (0.16-4.24)	5.18 (1.42-18.93)^a^
Laryngospasm	3 (2.4)	12 (9.7)	2 (1.6)	3.98 (1.08-14.76)	0.55 (0.09-3.41)	7.19 (1.56-33.24)^a^
Bronchospasm	0	1 (0.8)	1 (0.8)	NA	NA	1.65 (0.07-39.29)
Minor	50 (40.0)	69 (55.6)	28 (22.6)	1.97 (1.16-3.32)^a^	0.42 (0.24-0.74)^a^	4.71 (2.67-8.29)^a^
Desaturation	39 (31.2)	59 (47.6)	22 (17.7)	2.12 (1.24-3.62)^a^	0.46 (0.25-0.84)^a^	4.60 (2.55-8.33)^a^
Coughing	23 (18.4)	27 (21.8)	9 (7.3)	1.20 (0.61-2.34)	0.33 (0.14-0.78)^a^	3.60 (1.56-8.33)^a^
Airway obstruction	9 (7.2)	21 (16.9)	7 (5.6)	2.73 (1.18-6.30)	0.75 (0.27-2.12)	3.62 (1.48-8.88)^a^
Stridor (recovery)	5 (4.0)	6 (4.8)	2 (1.6)	1.43 (0.40-5.02)	0.42 (0.78-2.27)	3.38 (0.66-17.38)

Abbreviations: aOR, adjusted odds ratio; NA, not applicable; PRAE, perioperative respiratory adverse event.

^a^ *P* < .017.

^b^ Data are unadjusted OR (95% CI).

^c^ Values were adjusted for age, sex, American Society of Anesthesiologists physical status, body mass index, upper respiratory tract infection, passive smoking, and obstructive sleep apnea.

### Secondary Outcomes

Table 2 also details the frequency of the individual PRAEs among the 3 groups after adjustment. Intranasal midazolam for premedication was associated with a higher likelihood of desaturation compared with normal saline (aOR, 2.12; 95% CI, 1.24-3.62). The dexmedetomidine group had a lower incidence of desaturation (aOR, 0.46; 95% CI, 0.25-0.84) and coughing (aOR, 0.33; 95% CI, 0.14-0.78) compared with normal saline group. The incidences of laryngospasm (aOR, 7.19; 95% CI, 1.56-33.24), desaturation (aOR, 4.60; 95% CI, 2.55-8.33), coughing (aOR, 3.60; 95% CI, 1.56-8.33), and airway obstruction (aOR, 3.62; 95% CI, 1.48-8.88) were higher in the midazolam group than in the dexmedetomidine group.

Table 3 shows the occurrence of PRAEs during the induction and recovery periods. The differences were mainly manifested in the recovery period, and there was no significant difference among the 3 groups during the induction period. The overall incidence of URTI was 33.0% (123 of 373 children). We conducted a post hoc analysis of the incidence of PRAEs in children with URTIs in the past 4 weeks, and the rates were 39.5% (17 of 43 children) in the normal saline group, 64.4% (29 of 45 children) in the midazolam group, and 20.0% (7 of 35 children) in the dexmedetomidine group(eTable 2 in Supplement 2). Fifteen patients (12.1%) who received midazolam experienced nasal irritation and desaturation.

**Table 3.** Comparison of the Incidence of Each Individual Perioperative Respiratory Adverse Event Among the 3 Groups During the Induction and Recovery Period for Intention-to-Treat Analysis

Phase	Patients, No. (%)			aOR (95%CI)^a^		
	Normal saline (n = 125)	Midazolam (n = 124)	Dexmedetomidine (n = 124)	Midazolam vs normal saline	Dexmedetomidine vs normal saline	Midazolam vs dexmedetomidine
Induction (any)	20 (16)	26 (21.0)	18 (14.5)	1.44 (0.74-2.84)	0.84 (0.41-1.73)	1.95 (0.94-4.04)
Laryngospasm	2 (1.6)	7 (5.6)	1 (0.8)	3.46 (0.68-17.53)	0.43 (0.04-4.83)	6.48 (0.75-56.01)
Bronchospasm	0	0	0	NA	NA	NA
Desaturation	15 (12.0)	15 (12.1)	14 (11.3)	1.09 (0.49-2.43)	0.89 (0.39-2.00)	1.26 (0.54-2.93)
Coughing	5 (4.0)	10 (8.1)	2 (1.6)	2.56 (0.77-8.54)	0.45 (0.08-2.50)	10.11 (1.20-85.05)
Airway obstruction	5 (4.0)	8 (6.5)	4 (3.2)	1.44 (0.44-4.67)	0.75 (0.19-2.94)	2.71 (0.68-10.76)
Recovery (any)	49 (39.2)	70 (56.5)	24 (19.4)	1.94 (1.12-3.35)	0.34 (0.18-0.61)^b^	5.78 (3.16-10.58)^b^
Laryngospasm	1 (0.8)	5 (4.0)	1 (0.8)	4.05 (0.42-38.69)	0.77 (0.05-12.71)	6.48 (0.75-56.01)
Bronchospasm	0	1 (0.8)	1 (0.8)	NA	NA	1.51 (0.07-34.26)
Desaturation	36 (28.8)	55 (44.4)	16 (12.9)	1.97 (1.12-3.48)	0.34 (0.17-0.66)^b^	5.88 (3.02-11.43)^b^
Coughing	21 (16.8)	24 (19.4)	7 (5.7)	1.09 (0.54-2.22)	0.26 (0.10-0.67)^b^	4.17 (1.65-10.60)^a^
Airway obstruction	6 (4.8)	14 (11.3)	4 (3.2)	2.58 (0.92-7.25)	0.65 (0.17-2.42)	3.98 (1.23-12.91)
Stridor (recovery)	5 (4.0)	6 (4.8)	2 (1.6)	1.34 (0.38-4.74)	0.42 (0.08-2.27)	3.20 (0.62-16.45)

Abbreviations: aOR, adjusted odds ratio; NA, not applicable.

^a^ Values were adjusted for age, sex, American Society of Anesthesiologists physical status, body mass index, upper respiratory tract infection, passive smoking, and obstructive sleep apnea.

^b^ *P* < .017.

There was no significant difference in extubation time, time spent in the PACU after the extubation, or postoperative hospital stay among the 3 groups. Wong-Baker Pain Scale scores and Pediatric Anesthesia Emergency Delirium Scale scores were similar among groups, but fewer children in the dexmedetomidine group required postoperative analgesics than in the midazolam group. The rate of emergence delirium in the dexmedetomidine group was lower than those in the midazolam group and normal saline group (Table 4). Comparison of sedation success rates among the 3 groups (eFigure 2 in Supplement 2) and the heart rate values at different times among groups (eFigure 3 in Supplement 2) are shown in eAppendix 2 in Supplement 2.

**Table 4.** Comparison of Postoperative Nonrespiratory Adverse Events

Variable	Median (IQR)			*P* value
	Normal saline (n = 125)	Midazolam (n = 124)	Dexmedetomidine (n = 124)	
Extubation time, min	17.0 (12.0-23.0)	16.0 (13.0-20.0)	16.0 (12.3-22.0)	.65
Time spent in the postanesthesia care unit after the extubation, min	15.0 (12.0-17.0)	14.0 (12.0-16.0)	15.0 (12.0-17.0)	.46
Duration of postoperative hospital stay, d	2 (1-3)	2 (1-3)	2 (1-3)	.24
Wong-Baker Pain Scale score	2.0 (0-2.0)	2.0 (0-2.0)	2.0 (0-2.0)	.48
Children requiring analgesics, No. (%)	23 (18.4)	30 (24.2)	14 (11.3)^a^	.03
Pediatric Anesthesia Emergency Delirium Scale score	5.0 (2.0-9.0)	6.0 (3.0-10.0)	5.0 (2.0-8.0)	.14
Emergence delirium, patients, No. (%)	27 (21.6)	36 (29.0)	12 (9.7)^a^^,^^b^	.001
Vomiting, patients, No. (%)	1 (0.9)	4 (3.2)	2 (1.6)	.32

^a^ *P* < .017 vs the midazolam group.

^b^ *P* < .017 vs the normal saline group.

## Discussion

Tonsillectomy and adenoidectomy are among the most frequently performed surgical procedures in children.^1,20^ Direct trauma to the airway during the operation causes swelling of the upper respiratory tract and surrounding tissues in children; as a result, secretions are retained in the airway, thus greatly increasing the risk of PRAEs.

In this randomized clinical trial, children who were sedated with midazolam preoperatively had a higher risk of PRAEs compared with those sedated with dexmedetomidine and those in the saline control group. Previous studies were mostly observational, and the results were contradictory. The different routes of administration, sedation time, dose and depth of sedation, and intraoperative management strategies may all affect the outcome. In our study, the influence of confounding factors was effectively controlled. Compared with the normal saline control group, the midazolam group had a significantly increased risk of PRAEs, although previous studies^14,15,16^ have shown that midazolam has a protective effect on airway contraction. A number of vitro studies have shown that midazolam has a spasmolytic effect on histamine-induced bronchoconstriction, and it has been confirmed that midazolam has an in vitro bronchiectasis effect in animal experiments.^21,22,23^ Therefore, midazolam may have a protective effect against bronchospasm. However, midazolam was associated with increased incidence of desaturation and also was associated with a higher risk of laryngospasm and airway obstruction. The current research on the mechanism of midazolam mainly is focused on bronchospasm, and the association of midazolam with other adverse events is still unclear. This performance may be related to the paradoxical reaction of midazolam^24^; that is, the children show irritability and other behaviors that are completely opposite to sedation and hypnosis. Children with sympathetic nerve excitement and increased stress reaction may be responsible for the high incidence of PRAEs associated with midazolam; thus, more experiments are needed to further explore its mechanism.

Premedication with intranasal dexmedetomidine was associated with a significant decrease in the incidence of PRAEs, especially the incidence of oxygen desaturation and coughing. Several mechanisms may underlie this beneficial effect. First, dexmedetomidine may have increased the depth of anesthesia and, thus, reduced airway reflexes.^25,26^ Second, the direct airway smooth muscle effect of dexmedetomidine may have also contributed. Accordingly, it has been demonstrated that dexmedetomidine attenuates both exogenous acetylcholine-induced and C-fiber–mediated isolated tracheal ring contraction, suggesting that it has the effect of relaxing airway smooth muscle and suppressing cough.^27^ Finally, dexmedetomidine may have modulated the inflammatory process, which is associated with increased airway sensitivity; both interleukin-6 and tumor necrosis factor–α levels have been shown to be substantially reduced after the use of this drug.^28^ In our study, the dexmedetomidine group had less demand for fentanyl because of the mild analgesic effect.^29^ It is evident that intravenous fentanyl is associated with coughing and respiratory depression.^30^ Therefore, dexmedetomidine may also be associated with reduced incidence of coughing and desaturation by reducing the use of fentanyl.

There was no significant difference in the overall incidence of PRAEs in the 3 groups during the induction period. This was because preoxygenation was routinely used in our institution, which greatly decreased the incidence of oxygen desaturation, the most common PRAE, during the induction period. However, the midazolam group was more likely to experience laryngospasm and coughing. Owing to the use of tracheal tubes for airway management, almost no PRAEs were observed during the maintenance period.

We conducted a post hoc analysis in children with URTIs in the previous 4 weeks. The occurrence of URTI in the entire cohort was 33.0%, and the incidence of PRAEs in the midazolam group with URTI was 64.4% (vs 56.5% overall in that group), whereas the incidence of PRAEs in the dexmedetomidine group with URTI was 20.0% (vs 24.2% overall in that group). These findings may be associated with increased airway sensitivity and may be caused by potential chronic airway inflammation.

Our results show that dexmedetomidine facilitated tolerance of the endotracheal tube and significantly reduced coughing during extubation without affecting the extubation time, an effect possibly mediated via its sedative and analgesic properties. Consistent with research conducted in Turkey,^31^ dexmedetomidine can reduce the airway reflex and suppress a sharp increase in heart rate during extubation, which could be explained by the markedly decreased sympathetic activity.

Finally, we chose intranasal midazolam 0.1 mg/kg and dexmedetomidine 2.0 μg/kg for preoperative sedation. In previous studies,^32^ it was confirmed that 2.0 μg/kg dexmedetomidine may be a better choice than 1.0 μg/kg. The dose of intranasal midazolam is typically 0.2 mg/kg, but it has a burning sensation and will irritate the nasal cavity. In our pre-experiment, children refused nasal drops, and a large number of midazolam preparations leaked. Thus, we decided to try low-dose midazolam preparations, and the results showed that the onset time of a 0.1 mg/kg nasal drip was longer than that of a dose of 0.2 mg/kg; it took effect after approximately 10 to 15 minutes but could also achieve a satisfactory sedative effect after 30 minutes. It turns out that a reduction in the dose can relieve nasal irritation. In previous studies,^29^ it was reported that approximately 36.1% of patients receiving midazolam would have nasal discomfort and tearing, but in our study, only 15 patients (12.1%) who received midazolam exhibited the aforementioned symptoms.

In our study, the incidence of emergence delirium in the midazolam group was higher than that in the normal saline group, but the difference was not significant. The use of midazolam as pharmacological prevention for emergence delirium is controversial.^33,34^ Some studies have considered midazolam as a risk factor for emergence delirium,^35^ and others have considered midazolam to be useful as a pharmacological prevention strategy.^36^ Dexmedetomidine has been confirmed to have a preventive effect against emergence delirium,^37^ and the incidence in the dexmedetomidine group in our study was significantly lower than that in the midazolam and normal saline group. Therefore, dexmedetomidine may be a better choice than midazolam.

### Limitations

This study has limitations that should be considered. In this trial, the researchers were blinded to the treatments, but experienced anesthesiologists would easily be able to differentiate between the different sedatives by simply observing patient behavior, especially during the induction period. This might have led to investigator bias in which those diagnosing the outcome were aware of the group allocation and/or the study hypothesis. However, it is important to note that none of the anesthesiologists who participated in this study were aware of the study hypothesis; therefore, this risk of bias was reduced.

We did not routinely use antagonists because neostigmine causes gastrointestinal spasm, and the children often show persistent discomfort and crying. We did not monitor the train of 4 simulation, although the clinical manifestations provide a possible basis, we still cannot ensure that all children are completely unaffected by residual muscle relaxation.

In the process of the experiment, we found that the individual differences of the children themselves were also obvious, which may be related to the education level of the parents. In this experiment, the education level of the parents was not evaluated, so this factor may be ignored. Moreover, we acknowledge that the OSA status was assessed by the otolaryngologist from the clinical history rather than by polysomnography, which is the standard for diagnosis and quantitative description of OSA, and we did not grade the severity of OSA.

## Conclusions

The findings of this study suggest that both midazolam and dexmedetomidine can achieve satisfactory sedative effects intranasally before surgery, and dexmedetomidine may have a protective effect against the occurrence of PRAEs, whereas midazolam increases the risk of PRAEs during the perioperative period. Therefore, if there are no special contraindications, we recommend dexmedetomidine sedation before surgery for children undergoing tonsillectomy and adenoidectomy.

## Notes

Supplement 1.
Trial Protocol and Statistical Analysis Plan

Supplement 2. **eAppendix 1.** Supplemental Methods **eAppendix 2. **Supplemental Results **eTable 1.** Comparison of the Incidence of Each Individual Perioperative Respiratory Adverse Event (PRAE) Among the Three Groups Over the Perioperative Period for As-Per-Protocol Analysis **eTable 2.** Comparison of the Incidence of PRAEs in Each Group With Upper Respiratory Tract Infections **eFigure 1.** Comparison of Incidence of PRAEs Among the Three Groups **eFigure 2.** Sedation Success Rate Among the Three Groups **eFigure 3.** Heart Rate Values at Different Times

Supplement 3.
Data Sharing Statement
