# Supplementary material for: Effect of Intranasal Dexmedetomidine or Midazolam for Premedication on the Occurrence of Respiratory Adverse Events in Children Undergoing Tonsillectomy and Adenoidectomy: A Randomized Clinical Trial
Source: JAMA Netw Open. 2022 Aug 9;5(8):e2225473. doi: 10.1001/jamanetworkopen.2022.25473 (PMC9364121; doi:10.1001/jamanetworkopen.2022.25473)
Supplement: Supplement 1. — Trial Protocol and Statistical Analysis Plan [file jamanetwopen-e2225473-s001.pdf]

Clinical Study Protocol

TITLE: Effect of intranasal dexmedetomidine or midazolam for premedication on the occurrence of respiratory adverse events in children undergoing tonsill and adenoidectomy, a prospective, randomized, double-blind clinical trial.

PRINCIPAL INVESTIGATOR: Fangming Shen, MD, He Liu, PhD, Yueying Zhang, MD.

PROTOCOL SYNOPSIS

**Title:**	Effect of intranasal dexmedetomidine or midazolam for premedication on the occurrence of respiratory adverse events in children undergoing tonsill and adenoidectomy
**Study Type:**	A prospective, single-center, randomized, double-blind clinical trial
**Corresponding Author:**	YueYing Zhang, Jiangsu Province Key Laboratory of Anesthesiology, Xuzhou Medical University; Department of Anesthesiology, The Affiliated Hospital of Xuzhou Medical University, Xuzhou, Jiangsu, China. He Liu, Department of Anesthesiology, The Affiliated Huzhou Hospital, Zhejiang University School of Medicine || Huzhou Central Hospital, Huzhou 313003, China.
**Study Centres:**	The Children's Hospital of Xuzhou Medical University
**Ethics**	Approved by the Medical Ethics Committee of the Children's Hospital of Xuzhou Medical University (2020-05-01-H01)
**Trial Registration**	www.chictr.org.cn Identifiers: ChiCTR2000038359

CONTENTS

I. STUDY OBJECTIVES 3
II. BACKGROUND 4
A. Perioperative Respiratory Adverse Events 4
B. Sedative Premedication 4
C. Preliminary Studies 4
III. METHODS 6
A. Recruiting Methods 6
B. Inclusion Criteria 6
C. Exclusion Criteria 6
D. Consent Procedure 6
E. Randomization and Blinding 6
F. Sample Size Calculation 7
G. Statistical Analysis 7
H. The dose of Sedatives 7
I. Administration Method 7
J. Study Procedures 8
K. Surgical Method 10
L. Measurements and Endpoints 10
IV. PRE-EXPERIMENT OUTCOMES 14
V. RISKS 15
VI. DATE AND SAFETY MONITORING 16
VII. FUNDING 17
VIII. INFORMATION CONFIDENTIALITY 18
IX. LITERATURE CITED 18
Appendix 1 Definition Used For Risk Factors 19
Appendix 2 Wong-Baker Pain Scale 20
Appendix 3 Steward recovery score 21
Appendix 4 Definition Used for Respiratory Complications Recorded 22
Appendix 5 Funk Sedation Score. 23
Appendix 6: Paediatric Anesthesia Emergence Delirium Score 24

I. STUDY OBJECTIVES

Perioperative respiratory adverse events (PRAEs) are the most common complication during pediatric anesthesia, midazolam and dexmedetomidine have been commonly used in recent years as the most common preoperative sedatives in children, it may affect respiratory adverse events.

The aim of this prospective study was to investigate the effect of intranasal dexmedetomidine or midazolam used for premedication on the occurrence of respiratory adverse events.

II. BACKGROUND

A. Perioperative Respiratory Adverse Events

Perioperative respiratory adverse events (PRAEs) are the most common complication during pediatric anesthesia, manifested as minor adverse events (oxygen desaturation, airway obstruction, coughing, wheezing) and major adverse events (laryngospasm and bronchospasm). The occurrence of these complications can prolong hospitalization time, increase hospitalization costs, occupy medical resources, and bring varying degrees of physical and psychological damage to children.

The incidence of PRAEs is approximately 20%, but in some airway operations, such as tonsillectomy and adenoidectomy, the incidence is as high as 50%. Independent risk factors include age ≤ 6 years, American Society of Anesthesiologists (ASA) classification, recent upper respiratory tract infection(URI), lung disease, obesity, obstructive sleep apnea (OSA), passive smoking, and the use of endotracheal tube. These factors are very common in children undergoing tonsillectomy and adenoidectomy.

B. Sedative Premedication

Pediatric patients can experience signifificant anxiety and distress during the perioperative period. They are usually exceptionally uncooperative, fearful, anxious, or physically resistant, particularly during times of parental separation, venipuncture, or mask application. The use of sedative premedication may help to reduce their anxiety, minimize the emotional trauma, and facilitate a smooth induction of anesthesia. But there are no clear recommendations or well-documented clinical studies to guide us in choosing a certain sedative. Midazolam and dexmedetomidine, as the most common preoperative sedatives in children, have been commonly used in recent years, but their influence on PRAEs is still unclear.

C. Preliminary Studies

1. Midazolam

A prospective cohort study by Ungern-Sternberg included all children who had general anaesthesia for surgical or medical interventions, elective or urgent procedures at Princess Margaret Hospital for Children, Perth, Australia, and 9297 questionnaires were available for analysis, and the results showed that the risk of perioperative respiratory adverse events was higher in children premedicated with midazolam than in those not premedicated.

In Rampersad' stydy, based on a dataset of 335 patients, the author tried to explore the relationship between preoperative URI symptoms and adverse events during emergence from anesthesia, observed that the use of midazolam premedication seemed to increase postoperative respiratory complications (OR=3.05, P =.018).

However, there was a multicentre prospective observational cohort study of paediatric patients admitted to French paediatric tertiary care centres for a procedure requiring general anaesthesia and who presented with URTI or a history of URTI within the preceding 4 weeks, which reported that premedication with midazolam was protective against respiratory adverse events.

The results of these studies were contradictory.

2. Dexmedetomidine

A total of 134 children with CHD aged 0 to 16 years with recent URI undergoing interventional cardiac catheterisation. Children were randomised to receive either intranasal dexmedetomidine 1.5 µg/kg (DEX group) or intranasal saline (Placebo group) 30 to 45 min before anaesthesia induction. Administration of intranasal dexmedetomidine 1.5µg/kg 30 to 45 min before induction led to a reduction in the incidence of PRAE in children aged less than 3 years with recent URI undergoing interventional cardiac catheterisation.

A large number of studies on the effect of preoperative sedation have verified the efficacy and safety of sedatives, their impact on PRAEs has gradually become the focus of clinical attention. However, previous studies are mostly

observational, and there are still no randomized controlled trials with high-quality evidence.

III. METHODS

This prospective, single-center, randomized, double-blind clinical trial was carried out at the Children's Hospital of Xuzhou Medical University, Xuzhou, China from October 01, 2020 to June 30, 2021. The study protocol was approved by the Medical Ethics Committee of the Children's Hospital of Xuzhou Medical University (2020-05-01-H01), and was registered in the Chinese Clinical Trial Registration Center on September 21, 2020 (www.chictr.org.cn, ChiCTR2000038359). This report follows the Consolidated Standards of Reporting Trials (CONSORT) reporting guideline for randomized studies.

A. Recruiting Methods

Potential participants to the study were identified from the elective surgery list. Children 0 to 12 years old, with American Society of Anesthesiology (ASA) physical status (I&II) were eligible for inclusion if they were undergoing elective tonsillectomy with or without adenoidectomy. The study will involve the use of protected health information. The study site will gain permission from each subject to use their protected health information by written authorization. All subjects will be identified by the anesthesiologist at the subject's chosen treatment institution (The Children's Hospital).

B. Inclusion Criteria

Children 0 to 12 years old, with American Society of Anesthesiology (ASA) physical status (I&II) were eligible for inclusion if they were undergoing elective tonsillectomy with or without adenoidectomy.

C. Exclusion Criteria

1. Known cardiopulmonary diseases (uncorrected congenital heart disease, primary or secondary pulmonary hypertension, tumors, structural lung diseases);
2. Neuromuscular diseases;
3. BMI>30 kg/m^2;
4. Severe upper respiratory tract infection and the anesthesiologist recommends delaying surgery;
5. Allergy to either midazolam or dexmedetomidine;
6. Children whose parents refused to allow them to participate.

D. Consent Procedure

All potential subjects that are identified by the chief anesthetist and/or designee that meet the inclusion/exclusion criteria will be given the opportunity to participate. Parents/guardians/patients (when applicable) will be given the consent/assent during the screening visit. They will be given the opportunity to review the consent/assent and ask questions about the study. Parents/guardians/patients will be asked to summarize in their own words what participation in this research study involves and that they are comfortable with the risks and benefits of participating in the research study. Any additional questions they have will also be answered by the investigator prior to signing the consent/assent. Once the consent/assent form is signed, a signed and dated copy of the authorization form will be provided to the subject and another copy placed in the participant's medical record at The Children's Hospital.

E. Randomization and Blinding

The patients were randomised to the three study groups using computer-generated randomisation, with group allocation and study number concealed in sealed envelopes. On the day of the operation, the intranasal drugs were prepared in a 1-ml syringe by an anesthesia nurse who was not involved in the study, the active drug or placebo was administered by a fully trained anesthesiologist, all researchers directly involved in the study were blinded to the drug

being administered. Data are recorded by different observers independently in different period. The experimental results are analyzed by independent statisticians, and the grouping situation is hidden.

F. Sample Size Calculation

Our preliminary data suggested an approximate incidence of PRAEs in group N, M and D were 40%, 60% and 20%, respectively. The difference between groups reaches 20% was considered a clinically significant difference, after adjusting for multiplicity from making three pairwise comparisons, a sample size of 115 per group at a .017 two-sided significance level provided an 80% power to detect a 20% difference in the rate of PRAEs among the groups using Chi-Square Test. After allowing for 10% data loss due to unusable or missing data, we aimed to recruit 128 participants in each group, 384 cases in total.

G. Statistical Analysis

Data were analyzed using SPSS Statistics software version 26.0 (IBM, USA). The Kolmogorov-Smirnov test was used to determine whether the continuous data conform to the normal distribution. The quantitative variables that obey normal distribution are presented as mean ± SD, non-normal distribution data are represented by median (M) and interquartile range (IQR). Binomial variables are expressed as rate. The continuous data of normal distribution were analyzed by one-way analysis of variance (ANOVA), the continuous data of non-normal distribution among the three groups were analyzed by the Kruskal–Wallis rank-sum test. Categorical data were analyzed using the Chi-square test, the *P* value is adjusted according to Bonferroni method and fixed at .017 for pairwise comparison. $P<.05$ was considered to indicate statistical significance.

Outcome analyses were performed in the intention-to-treat population, a per-protocol analysis was also performed for the primary endpoint. Primary outcome was analyzed using Chi-square test or Fisher exact test, the crude odds ratio (OR) and 95% CI reported were calculated. Adjusted odds ratio (aOR) and 95% CI were calculated for both primary and secondary outcomes. Age, sex, American Society of Anesthesiologists physical status, BMI, URI, passive smoking and OSA were adjusted for using binary logistic regression, age and BMI adjusted in the models as continuous variables.

H. The dose of Sedatives

According to the previous studies and the "Chinese Anesthesiology Guidelines and Expert Consensus", the recommended doses of sedatives were dexmedetomidine 2.0 μg/kg and midazolam 0.2 mg/kg for sedation. In a review which concluded that a dose of 2.0 μg/kg is the optimum choice because higher dexmedetomidine doses do not necessarily increase the rate of successful sedation but may cause severe bradycardia, and 2.0 μg/kg takes effect faster than 1.0 μg/kg, we finally chose 2.0 μg/kg.

When intranasal midazolam 0.2 mg/kg in our pre-experiment,we found that it has a burning sensation and will irritate the nasal cavity, children refused nasal drops, and a large number of midazolam preparations leaked. Thus, the researchers decided to try low-dose midazolam preparations, and the results showed that the onset time of a 0.1 mg/kg nasal drip was longer than that of 0.2 mg/kg, it took effect after approximately 10-15 minutes but could also achieve a satisfactory sedative effect after 30 minutes. We finally chose dexmedetomidine 2.0 μg/kg and midazolam 0.1 mg/kg for sedation.

I. Administration Method

According to previous studies, intranasal premedication is more acceptable to children than oral and intravenous methods. In our pre-experiment, we compared the use of nose drops and atomiser, and found that more children tend to reject atomiser, and their compliance with nose drops will be better. Li et al (2016) compared intranasal delivery of dexmedetomidine either by drops from a syringe or an atomiser in children undergoing transthoracic echocardiography.

The successful sedation rate was similar at 82.5% and 84.5% for atomiser and drops, respectively. For the above reasons, we finally chose the nasal administration method.

J. Study Procedures

1. Preoperative assessment

During the preoperative assessment, we collected baseline data include demographic characteristics, past history, laboratory and other examinations, as well as common risk factors for PRAEs: including respiratory tract infection within the past 4 weeks, previous asthma or active asthma, nocturnal dry cough, past/present eczema, history of allergies, preterm birth, obstructive sleep apnea (OSA), passive smoking, detailed definitions of risk factors are shown in Appendix 1. And obtained the informed consent of their parents or guardians if eligible.

We will provide a video for the children to let them see everything that he/she will go through during the operation, parents will introduce each process one by one, allowing the parent to simulate the nasal drip process, so the parents or guardians would make psychological preparations for better cooperation. All patients fasted 8 hours for solids and 2 h for clear liquids.

Appendix 1 Definition Used For Risk Factors.

Risk factors	Brief Definition Applied in This Study
Upper respiratory infections	Signs of runny nose, cough and/or fever (＞38℃) but deemed fit for anesthesia by independent consultant anesthesiologist.
Asthma	More than three episodes of wheezing experienced during the past year.
Allergy	Allergies to pollen, food, or medications.
Past/Present eczema	Persistent eczema observed in past or currently.
Passive smoking	Child exposed to parents/ caretakers smoking independent of location, e.g. inside or outside of house.
OSA	Performed by the otolaryngologist based on clinical criteria such as medical history, symptoms, signs, and questionnaire (OSA-18).
Preterm delivery	Delivery between 28 gestational weeks (196 days) and 37 gestational weeks (259 days).

OSA=Obstructive Sleep Apnea；

2. Management of premedication

Children received intranasal premedication in the preoperative holding area at approximately 30-60 min before induction of anesthesia in the presence of one parent in a full resuscitation facility. Children received intranasal midazolam 0.1mg/kg (up to a maximum 5 mg) or dexmedetomidine 2.0 μg/kg (up to a maximum 100 μg), intranasal midazolam was prepared from a (5 mg/ml) parenteral preparation, while dexmedetomidine was prepared from the (100 μg/ml) parenteral preparation, with 0.9% saline added to make a final volume of 1 ml, the control group was given 1 ml 0.9% saline. The prepared drug solution was administered cautiously in both nostrils using a needleless 1 ml syringe as drop by drop to avoid wastage of the drug through anterior and posterior nostrils, 0.15-0.2ml for each administration, pinch the nostril after administration, re-administer at 10-15s intervals, and finish the administration within 2-3min. The child was positioned on the parent's lap in a recumbent position during administration and was allowed to sit up or assume a more comfortable position 5 min later. Children who failed to take the premedication were excluded from the study protocol. Haemodynamic parameters [heart rate (HR) and oxygen saturation] were continuously monitored.

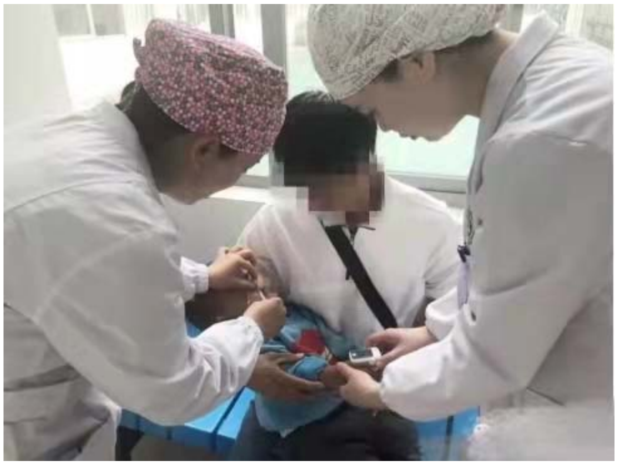

After administration, if the child had insufficient sedation, expressing anxiety and fear, etc., the parents will give psychological comfort and conduct behavioral intervention through distracting methods such as watching cartoons and playing with toys. If it was still not enough, we will add sedatives, and this patient will be excluded from analysis.

3. Management of general anesthesia

When entering the surgery room, routine physiological monitoring included electrocardiography, noninvasive blood pressure, capnography, and pulse oximetry. The type of anesthesia induction is determined by the responsible anesthetist independently. Preoxygenation was routinely used, **intravenous induction** was achieved with dexamethasone 0.1mg/kg, etomidate 0.3mg/kg, fentanyl 1-2 μg/kg, cisatracurium 0.15mg/kg after opening the intravenous access. **Inhalation induction** was carried out with sevoflurane, 8% sevoflurane with 100% oxygen 3-6 L/min, after pre-filling the circuit, put the mask on the face of the child quickly, when consciousness disappears, adjust sevoflurane to 3%-4%, 100% oxygen to 1-2L/min, maintain spontaneous breathing, assist breathing when necessary, then opening the intravenous access and give auxiliary drugs. Airway management was performed with a endotracheal tube in all children, and lidocaine cream is applied to the cuff. Anesthesia maintenance: sevoflurane 1% continuous inhalation, propofol 2-4 ($mg \cdot kg^{-1} \cdot h^{-1}$), remifentanil 10-18 ($\mu g \cdot kg^{-1} \cdot h^{-1}$), ventilation was controlled to maintain an endtidal carbon dioxide partial pressure ($PetCO_2$) of 35–45 mmHg, use entropy index monitoring during the operation, keep the entropy index between 40-60, and added muscle relaxant according to surgical needs. After the end of surgery, secretions and intraoperative irrigation fluid in mouth were sucked out to avoid aspiration. In order to improve the efficiency of surgery and speed up the turnaround, all surgical patients were transferred to the PACU for extubation in our institution.

The criteria used to ascertain whether reversal agents were administered: If the patient have autonomous breathing but the time less than 45 minutes from the last last time of administration, OR when the anesthesiologist thinks that the patient had residual muscle relaxant (There may be a residual muscle relaxant if any term is violated: Awareness, choking and swallowing reflex recovery; Raise head more than 5s from the pillow; Breathing is stable and tidal volume, breathing frequency returns to normal), we will consider the use of neostigmine 0.04～0.07mg/kg, concomitant use with atropine.

Tracheal extubation was undertaken by a specialized pediatric anesthesiologist in PACU when the child had demonstrated facial grimacing, adequate tidal volumes and respiratory rate, coughing with an open mouth or opening of their eyes and purposeful movements.

After extubation, postoperative pain was assessed using Wong-Baker Pain Scale (Appendix 2, Supplement 1), fentanyl (0.5-1.0 μg/kg) were administered if the score was more than 4 for pain management. Return to the ward when steward score (Appendix 3, Supplement 1) >4. Metoclopramide will be given if the child presented nausea and vomiting. The anestheic management was performed according to "Chinese Anesthesiology Guidelines and Expert Consensus" and there was no deviation.

Appendix 2 Wong-Baker Pain Scale

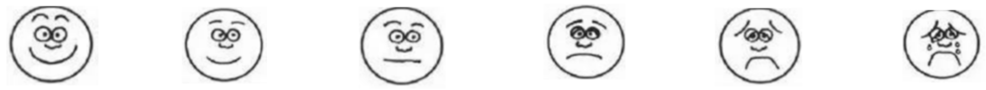

0	2	4	6	8	10
No Hurt	Hurts Little Bit	Hurt Little More	Hurt Even More	Hurt Whole Lot	Hurt Worst

Appendix 3 Steward recovery score

Clinical features	Score
Consciousness	
Awake	2
Responding to stimuli	1
Not responding	0
Airway	
Coughing on command or crying	2
Maintaining good airway	1
Airway requires maintenance	0
Movement	
Moving limbs purposefully	2
Nonpurposeful movement of limbs	1
Not moving	0

4. Postoperative Analgesia

All patients used tracheal tubes for airway management, and the cuff was infiltrated with lidocaine cream to relieve postoperative sore throat. After extubation, postoperative pain was assessed using *Wong-Baker Pain Scale* (Appendix 3), fentanyl (0.5-1.0 μg/kg) were administered if the score was more than 4 for pain management, no other analgesics were used.

K. Surgical Method

Coblation tonsillectomy with or without adenoidectomy.

L. Measurements and Endpoints

After intranasal drug administration, the heart rate, pulse oxygen saturation were recorded every 5 minutes, and the duration of sedation was recorded, and the Sedation success rate was recorded when entering the operating room. Intraoperative data was recorded by another investigator, included type and duration of anesthesia, type and doses of anesthetics and other medications, surgery duration, volume of estimated bleeding, fluid balance, and vital signs (heart rate, blood pressure, and pulse oximetry). After entering the PACU, an independent nurse who was blinded to the premedication drug was responsible for observing and recording PRAEs, extubation time (the time from the end of surgery to tracheal extubation), the time spent in the PACU after the extubation.

The diagnosis of respiratory adverse events and risk factors are known to be operator dependent owing to the composite aspect of such adverse events and the clinical judgment process involved. To limit reporting errors, clear definitions were provided to the researchers. We conducted special training for evaluators before the start of the trial, and the reliability of our outcome data collection form for capturing the perioperative date was also assessed.

The primary outcome was the difference in the incidence of PRAEs among the three groups.

The secondary outcomes were the frequency of the individual respiratory adverse events. Furthermore, in line with clinical importance, these perioperative respiratory adverse events were clustered into two groups; major (bronchospasm and laryngospasm) and minor (all other respiratory adverse events) respiratory adverse events (**Appendix 4** shows details and definitions); The incidence of respiratory adverse events in induction period and recovery period (the time from the end of surgery to discharge from PACU).

Other secondary outcomes include the the level of sedation (*FUNK score,* **Appendix 5**), nausea, vomiting and other adverse events, postoperative pain was assessed using *Wong-Baker Pain* Scale, postoperative emergence delirium was assessed using *PAED scale*(Appendix 6).

Appendix 4 Definition Used for Respiratory Complications Recorded

Respiratory Adverse Events	Definition
Laryngospasm	Complete airway obstruction with associated muscle rigidity of the abdominal and chest walls.
Bronchospasm	Increased respiratory effort, particularly during expiration and wheeze on auscultation.
Oxygen desaturation	Less than 95% more than 10s, or less than 90%.
Coughing	A series of pronounced, persistent severe coughs lasting more than 10s.
Airway obstruction	Presence of airway obstruction in combination with a snoring noise and/or respiratory efforts.
Stridor(recovery)	High-pitched sound during breathing in the postoperative period.

Appendix 5 Funk Sedation Score.

Score	Anxiolysis	Separation	Puncture	Mask induction
1	Panicky	Combative,clinging	Fight w/o success	Combative
2	Moaning	Anxious,consolable	Fight with success	Resistance
3	Composed	Calm	Minor resistance	Minor resistance
4	Friendly	Sleeping	No reaction	Unafraid,cooperative

A score of 3 or 4 is considered as successful sedation, and a total score of ≥12 is considered clinically effective sedation

Appendix 6: Paediatric Anesthesia Emergence Delirium Score

**Behavior**	**Not at all**	**Just a bit**	**Quite a bit**	**Very much**	**Extremely**
Makes eye contact with care giver	4	3	2	1	0
Purposeful actions	4	3	2	1	0
Aware of surroundings	4	3	2	1	0
Restless	0	1	2	3	4
Inconsolable	0	1	2	3	4

A total PAED scores ≥ 10 were considered indicative of the presence of ED

IV. PRE-EXPERIMENT OUTCOMES

We observed 30 children in our pre-experiment, when midazolam 0.1 mg/kg, dexmedetomidine 2.0 µg/kg, and saline control group were sedated before surgery, the incidence of PRAEs was 60%, 20%, 40% respectively.

Characteristics of the patients at baseline and the difference in the rate of occurrence of PRAEs in pre-experiment

		N（n=10）	M（n=10）	D（n=10）
Baseline	Male/Female	7/3	5/5	8/2
	Age, mean±SD	9.70±1.16	7.00±2.87	7.80±3.12
	Height, mean±SD	145.40±8.64	133.30±21.37	136.20±21.52
	Weight, mean±SD	41.20±9.63	32.45±18.93	37.00±20.27
	ASA(I/II)	4/6	3/7	2/8
Risk factors	Upper Respiratory Infections	4（40%）	5（50%）	3（30%）
	Allergy	3（30%）	1（10%）	0
	Past/Present eczema	1（10%）	1（10%）	1（10%）
	Passive smoking	5（50%）	6（60%）	4（40%）
	OSA	9（90%）	7（70%）	10（100%）
	Preterm delivery	1（10%）	1（10%）	0
**Outcomes**	**PRAEs**	**4（40%）**	**6（60%）**	**2（20%）**

V. RISKS

A. Subjects

A serious potential risk for the subjects are the bradycardia and hypoxia , and the dose is within the normal allowable range, so the incidence of side effects is extremely low. There are few reports of mild adverse events such as hypotension and respiratory depression in previous studies, and no serious adverse events for clinical reports of complications, we routinely monitor the heart rate, oxygen saturation after administration of sedatives. If there are discomforts such as decreased oxygen saturation, bradycardia, hypotension, dizziness, etc., symptomatic treatment such as oxygen inhalation should be performed immediately. In addition, preoperative sedation may also have poor or even ineffective sedation, that is, anxiety, tension and other emotions may still occur after administration.

B. Investigators/Institutions

There are no known risks to investigators.

VI. DATE AND SAFETY MONITORING

Clinical research will formulate a corresponding data safety monitoring plan according to the size of the risk. In the implementation stage of clinical research, record all adverse events in detail, handle and track them properly until they are properly resolved or the condition is stable, and report serious adverse events and unexpected events to the ethics committee, competent authoritiesn and drug regulatory authorities in a timely manner as required; The principal investigator will periodically conduct a cumulative review of all adverse events, and if necessary, hold an investigator meeting to assess the risks and benefits of the study; This experiment is a double-blind trial, and the blinding is urgently opened if necessary to ensure the safety and rights of the subjects; We will arrange independent data monitors to monitor the research data, and high-risk studies will establish an independent data safety monitoring committee to monitor the accumulated safety data and efficacy data to make a decision on whether to continue the study.

VII. FUNDING

This research did not receive any specific grant from funding agencies in the public, commercial, or not-for-profit sector.

VIII. INFORMATION CONFIDENTIALITY

Medical records will be kept in the hospital, the investigators and ethics committees will be allowed to access the patient's medical records. Any public reporting of the results of this study will not disclose the patient's personal identity.

IX. LITERATURE CITED

1. von Ungern-Sternberg BS, Boda K, Chambers NA, et al. Risk assessment for respiratory complications in paediatric anaesthesia: a prospective cohort study. *Lancet (London, England)* 2010; **376**: 773-83
2. Rachel Homer J, Elwood T, Peterson D, Rampersad S. Risk factors for adverse events in children with colds emerging from anesthesia: a logistic regression. *Paediatric anaesthesia* 2007; **17**: 154-61
3. Michel F, Vacher T, Julien-Marsollier F, et al. Peri-operative respiratory adverse events in children with upper respiratory tract infections allowed to proceed with anaesthesia: A French national cohort study. *European journal of anaesthesiology* 2018; **35**: 919-28
4. Zhang S, Zhang R, Cai M, Zhang K, Zhang M, Zheng J. Intranasal dexmedetomidine premedication in children with recent upper respiratory tract infection undergoing interventional cardiac catheterisation: A randomised controlled trial. *European journal of anaesthesiology* 2020; **37**: 85-90
5. Liu S, Wang Y, Zhu Y, Yu T, Zhao H. Safety and sedative effect of intranasal dexmedetomidine in mandibular third molar surgery: a systematic review and meta-analysis. *Drug Des Devel Ther* 2019; **13**: 1301-10

Appendix 1 Definition Used For Risk Factors.

Risk factors	Brief Definition Applied in This Study
Upper respiratory infections	Signs of runny nose, cough and/or fever (＞38℃) but deemed fit for anesthesia by independent consultant anesthesiologist.
Asthma	More than three episodes of wheezing experienced during the past year.
Allergy	Allergies to pollen, food, or medications.
Past/Present eczema	Persistent eczema observed in past or currently.
Passive smoking	Child exposed to parents/ caretakers smoking independent of location, e.g. inside or outside of house.
OSA	Performed by the otolaryngologist based on clinical criteria such as medical history, symptoms, signs, and questionnaire (OSA-18).
Preterm delivery	Delivery between 28 gestational weeks (196 days) and 37 gestational weeks (259 days).

Appendix 2 Wong-Baker Pain Scale

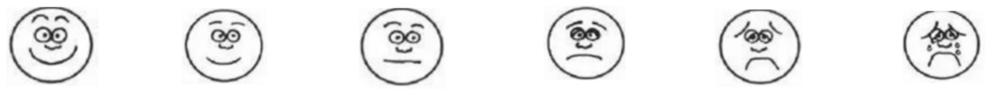

0	2	4	6	8	10
No	Hurts	Hurt	Hurt	Hurt	Hurt
Hurt	Little Bit	Little More	Even More	Whole Lot	Worst

Appendix 3 Steward recovery score

Clinical features	Score
Consciousness	
Awake	2
Responding to stimuli	1
Not responding	0
Airway	
Coughing on command or crying	2
Maintaining good airway	1
Airway requires maintenance	0
Movement	
Moving limbs purposefully	2
Nonpurposeful movement of limbs	1
Not moving	0

Appendix 4 Definition Used for Respiratory Complications Recorded

Respiratory Adverse Events	Definition
Laryngospasm	Complete airway obstruction with associated muscle rigidity of the abdominal and chest walls.
Bronchospasm	Increased respiratory effort, particularly during expiration and wheeze on auscultation.
Oxygen desaturation	Less than 95% more than 10s, or less than 90%.
Coughing	A series of pronounced, persistent severe coughs lasting more than 10s.
Airway obstruction	Presence of airway obstruction in combination with a snoring noise and/or respiratory efforts.
Stridor(recovery)	High-pitched sound during breathing in the postoperative period.

Appendix 5 Funk Sedation Score.

Score	Anxiolysis	Separation	Puncture	Mask induction
1	Panicky	Combative,clinging	Fight w/o success	Combative
2	Moaning	Anxious,consolable	Fight with success	Resistance
3	Composed	Calm	Minor resistance	Minor resistance
4	Friendly	Sleeping	No reaction	Unafraid,cooperative

A score of 3 or 4 is considered as successful sedation, and a total score of ≥12 is considered clinically ueffective sedation.

Appendix 6: Paediatric Anesthesia Emergence Delirium Score

**Behavior**	**Not at all**	**Just a bit**	**Quite a bit**	**Very much**	**Extremely**
Makes eye contact with care giver	4	3	2	1	0
Purposeful actions	4	3	2	1	0
Aware of surroundings	4	3	2	1	0
Restless	0	1	2	3	4
Inconsolable	0	1	2	3	4

A total PAED scores ≥ 10 were considered indicative of the presence of ED
