# Supplementary material for: Effect of Intranasal Dexmedetomidine or Midazolam for Premedication on the Occurrence of Respiratory Adverse Events in Children Undergoing Tonsillectomy and Adenoidectomy: A Randomized Clinical Trial
Source: JAMA Netw Open. 2022 Aug 9;5(8):e2225473. doi: 10.1001/jamanetworkopen.2022.25473 (PMC9364121; doi:10.1001/jamanetworkopen.2022.25473)
Supplement: Supplement 2. — eAppendix 1. Supplemental Methods eAppendix 2. Supplemental Results eTable 1. Comparison of the Incidence of Each Individual Perioperative Respiratory Adverse Event (PRAE) Among the Three Groups Over the Perioperative Period for As-Per-Protocol Analysis eTable 2. Comparison of the Incidence of PRAEs in Each Group With Upper Respiratory Tract Infections eFigure 1. Comparison of Incidence of PRAEs Among the Three Groups eFigure 2. Sedation Success Rate Among the Three Groups eFigure 3. Heart Rate Values at Different Times [file jamanetwopen-e2225473-s002.pdf]

Supplemental Online Content

Shen F, Zhang Q, Xu Y, et al. Effect of intranasal dexmedetomidine or midazolam for premedication on the occurrence of respiratory adverse events in children undergoing tonsillectomy and adenoidectomy: a randomized clinical trial. *JAMA Netw Open*. 2022;5(8):e2225473. doi:10.1001/jamanetworkopen.2022.25473

eAppendix 1. Supplemental Methods
eAppendix 2. Supplemental Results
eTable 1. Comparison of the Incidence of Each Individual Perioperative Respiratory Adverse Event (PRAE) Among the Three Groups Over the Perioperative Period for As-Per-Protocol Analysis
eTable 2. Comparison of the Incidence of PRAEs in Each Group With Upper Respiratory Tract Infections
eFigure 1. Comparison of Incidence of PRAEs Among the Three Groups
eFigure 2. Sedation Success Rate Among the Three Groups
eFigure 3. Heart Rate Values at Different Times

This supplemental material has been provided by the authors to give readers additional information about their work.

© 2022 Shen F et al. *JAMA Network Open.*

eAppendix 1. Supplemental Methods

Further detail on statistical analyses

Primary outcome was analyzed using Chi-square test or Fisher exact test, the crude odds ratio (OR) and 95% CI reported were calculated.

Adjusted odds ratio (aOR) and 95% CI were calculated for both primary and secondary outcomes. Age, sex, American Society of Anesthesiologists physical status, Body Mass Index(BMI), Upper Respiratory Infection(URI), passive smoking and Obstructive Sleep Apnea(OSA) were adjusted for using binary logistic regression, age and BMI adjusted in the models as continuous variables.

Intention-to-treat analysis AND as-per-protocol analysis

Outcome analyses were performed in the intention-to-treat population, a per-protocol analysis was also performed for the primary endpoint.

We excluded 33 cases in as-per-protocol analysis: 10 patients were preoperatively sedated for less than 30 minutes, 6 patients were preoperatively sedated for more than 60 minutes, 8 patients still accepted intravenous midazolam during the anesthesia induction period, 9 patients refused a preoperative nasal drip(figure 1), these cases violated the protocol of the trial, excluding them would destroy the balance among groups and be inconsistent with actual clinical practice, but the intention-to-treat analysis may underestimate the efficacy and increase the possibility of type II error, so we reported the ITT in the main results, and the PP in the eResult.

Post hoc analysis

We conducted a post hoc analysis for children with upper respiratory tract infections(URI) in the previous 4 weeks, previous studies have found that approximately 30% of children undergoing elective surgery have active URI symptoms. URI is associated with an increased risk for PRAEs, the most common reason for delaying surgery in children is due to upper respiratory tract infection. The incidence of PRAEs was reported to be 2-3 times higher in children with URTI than without URTI.

© 2022 Shen F et al. *JAMA Network Open.*

eAppendix 2. Supplemental Results

As-per-protocol analysis

The incidence of PRAEs among the three groups are shown in **eTable 1**, the crude and adjusted OR were presented. Consistent with the intention-to-treat analysis, children in midazolam group were more likely to experience PRAEs than those in normal saline group after adjusting for age, sex, ASA, BMI, OSA, URI and passive smoking (65/112, [58.0%] vs. 48/114, [42.1%]; aOR:1.92; 95%CI, 1.11-3.33), while dexmedetomidine group had a significantly lower PRAEs incidence than normal saline group(27/114, [23.7%] vs. 48/114, [42.1%]; aOR:0.39; 95%CI,0.22-0.70). Compared with dexmedetomidine group, midazolam group had a higher risk of PRAEs (aOR: 4.95; 95%CI:2.74-8.93)

© 2022 Shen F et al. *JAMA Network Open.*

**eTable 1.** Comparison of the Incidence of Each Individual Perioperative Respiratory Adverse Event (PRAE) Among the Three Groups Over the Perioperative Period for As-Per-Protocol Analysis							
Perioperative respiratory adverse events		N (n=114)	M (n=112)	D (n=114)	M *vs* N	D *vs* N	M *vs* D
					**OR/95CI**	**OR/95CI**	**OR/95CI**
**Any-unadjusted**		48(42.1)	65(58.0)	27(23.7)	1.90(1.12-3.23)*	0.43(0.24-0.75)*	4.46(2.52-7.90)*
					**aOR/95CI**	**aOR/95CI**	**aOR/95CI**
**Any-adjusted**		48(42.1)	65(58.0)	27(23.7)	1.92(1.11-3.33)	0.39(0.22-0.70)*	4.95(2.74-8.93)*
	Ⅰ. Laryngospasm	3(2.6)	10(8.9)	2(1.8)	3.12(0.81-11.94)	0.55(0.09-3.38)	5.72(1.21-27.11)
	Ⅱ. Bronchospasm	0	1(0.9)	1(0.9)	-	-	1.51(0.07-34.26)
Major (I&II)		3(2.6)	11(9.8)	3(2.6)	3.36(0.89-12.72)	0.83(0.16-4.27)	4.07(1.09-15.25)
	Ⅲ. Desaturation	37(32.5)	55(49.1)	21(18.4)	2.06(1.18-3.60)*	0.43(0.23-0.81)*	4.78(2.58-8.87)*
	Ⅳ. Coughing	22(19.3)	25(22.3)	8(7.0)	1.18(0.59-2.35)	0.29(0.12-0.71)*	4.09(1.69-9.91)*
	Ⅴ.Airway obstruction	9(7.9)	21(18.8)	6(5.3)	2.58(1.11-6.00)	0.62(0.21-1.82)	4.18(1.61-10.86)*
	Ⅵ. Stridor (recovery)	5(4.4)	6(5.4)	2(1.8)	1.34(0.38-4.74)	0.42(0.08-2.27)	3.20(0.62-16.45)
Minor (III-VI)		47(41.2)	64(57.1)	26(22.8)	1.91(1.11-3.31)	0.38(0.21-0.69)*	5.07(2.80-9.18)*
Data are number of patients (%). Adjusted values are for age, sex, ASA physical status, BMI, URI, passive smoking and OSA; OR=odds ratio; aOR=adjusted odds ratio. N=Normal saline. M=Midazolam. D=Dexmedetomidine. *$P < .017$.							

© 2022 Shen F et al. *JAMA Network Open.*

Post hoc analysis

The occurrence of URI was 33.0%, the incidence of PRAEs in midazolam group was 64.4% (vs. 56.5% in general population), while dexmedetomidine was 20.0% (vs. 24.2% in general population). The risk of PRAEs in the midazolam group was higher than that in the control group (aOR:3.49, 95%CI:1.28-9.47), and the dexmedetomidine group had a lower occurrence of PRAEs during the perioperative period (aOR:0.25, 95%CI:0.08-0.77), the difference was more obvious between dexmedetomidine group and midazolam group(aOR:14.22, 95%CI:4.16-48.56), eTable 2.

© 2022 Shen F et al. *JAMA Network Open.*

eTable 2. Comparison of the Incidence of PRAEs in Each Group With Upper Respiratory Tract Infections.							
Perioperative respiratory adverse events		N (n=43)	M (n=45)	D (n=35)	M *vs* N aOR(95%CI)	D *vs* N aOR(95%CI)	M *vs* D aOR(95%CI)
**Any-adjusted**		17(39.5)	29(64.4)	7(20.0)	3.49(1.28-9.47)*	0.25(0.08-0.77)*	14.22(4.16-48.56)*
	Laryngospasm	1(2.3)	4(8.9)	0	5.38(0.33-87.42)	-	-
	Bronchospasm	0	0	0	-	-	-
	Desaturation	14(32.6)	22(48.9)	5(14.3)	2.56(0.96-6.78)	0.36(0.11-1.21)	7.14(2.11-24.15)*
	Coughing	13(30.2)	18(40.0)	2(5.7)	1.84(0.68-4.97)	0.10(0.02-0.51)*	18.36(3.53-95.44)*
	Airway obstruction	3(7.0)	7(15.6)	3(8.6)	1.89(0.41-8.75)	1.03(0.18-5.75)	1.84(0.39-8.75)
	Stridor (recovery)	2(4.7)	1(2.2)	1(2.9)	1.54(0.09-25.98)	0.44(0.02-7.93)	3.51(0.11-109.10)

Data are number of patients (%). Adjusted values are for age, sex, ASA physical status, BMI, passive smoking and OSA; aOR=adjusted odds ratio. N=Normal saline. M=Midazolam. D=Dexmedetomidine. *P＜.017.

© 2022 Shen F et al. *JAMA Network Open.*

Secondary outcomes

Sedation success rate

FUNK score was recorded when entering the operating room, it assess sedative effects in anxiolysis, separation, puncture, mask induction, the score of 1 or 2 is considered ineffective sedation, whlie a score of 3 or 4 is considered effective sedation, total funk score ≥12 is considered clinically effective sedation.

Success rates for anxiolysis, separation, puncture, mask induction and clinically effective sedation before induction of anesthesia among groups are shown in eFigure 2. Both dexmedetomidine group and midazolam group had higher success rates in anxiolysis, separation and mask induction compared normal saline group, dexmedetomidine may be better than midazolam in reducing anxiety. There was no significant difference in the success rate of clinical effective sedation between dexmedetomidine and midazolam group (68.5% vs. 77.4%, $P > .017$).

Heart rate values at different times

Changes in heart rate were recorded at ten points [T_0, preadministration of premedication (baseline); T_1, 5 minutes after administration; T_2, 10 minutes after administration; T_3, 15 minutes after administration; T_4, 20 minutes after administration; T_5, 25 minutes after administration; T_6, 30 minutes after administration; T_7, after induction; T_8, after successful endotracheal tube insertion; T_9, after endotracheal tube removal].

The heart rate of dexmedetomidine group and midazolam group was slightly lower than that of normal saline group after sedation, but no bradycardia was observed. At the time of extubation (T_9), the heart rate of dexmedetomidine group was lower than that of normal saline group and midazolam group ($P<.017$). There were no significant difference between the groups in other moments ($P>.017$).

When comparing within group, compared with the baseline value (T_0), the heart rates of the three groups significantly decreased after induction (T_7) and after intubation (T_8) ($P<.001$), and the heart rates of normal saline and midazolam group significantly decreased after extubation (T_9) than the baseline value (T_0) ($P < .001$), whlie dexmedetomidine can suppress a sharp increase in heart rate during extubation..

© 2022 Shen F et al. *JAMA Network Open.*

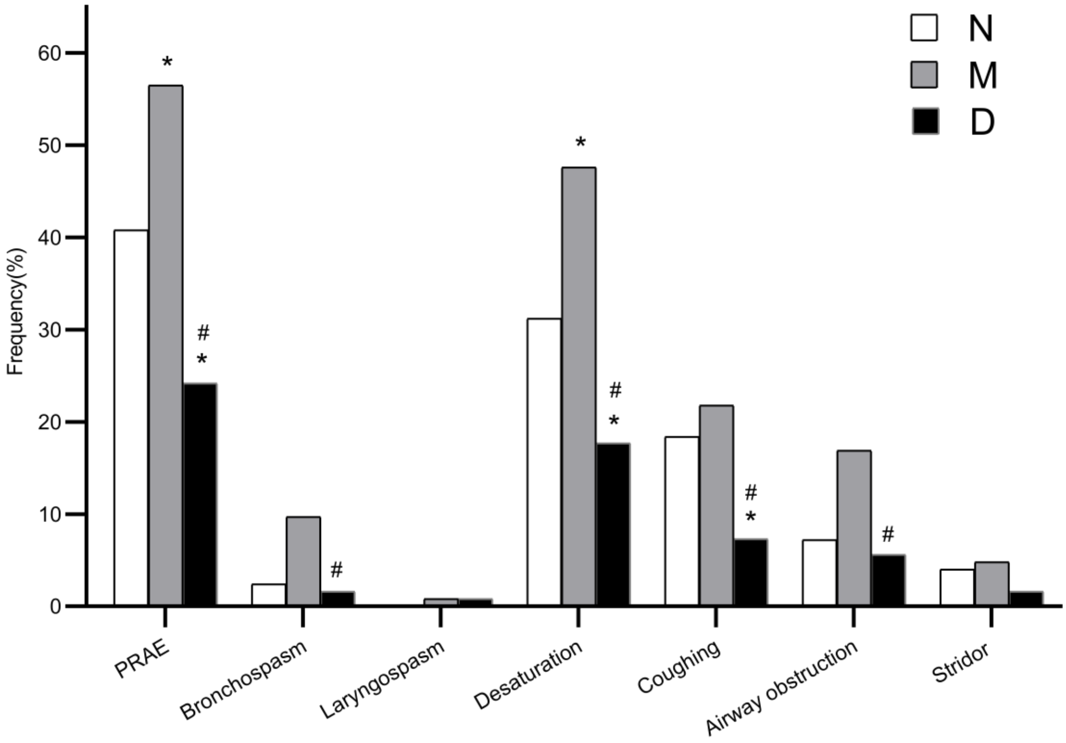

eFigure 1. Comparison of Incidence of PRAEs Among the Three Groups

[*]*P*＜.017, compared with Normal saline group；[#]*P*＜.017, compared with Midazolam group.

© 2022 Shen F et al. *JAMA Network Open.*

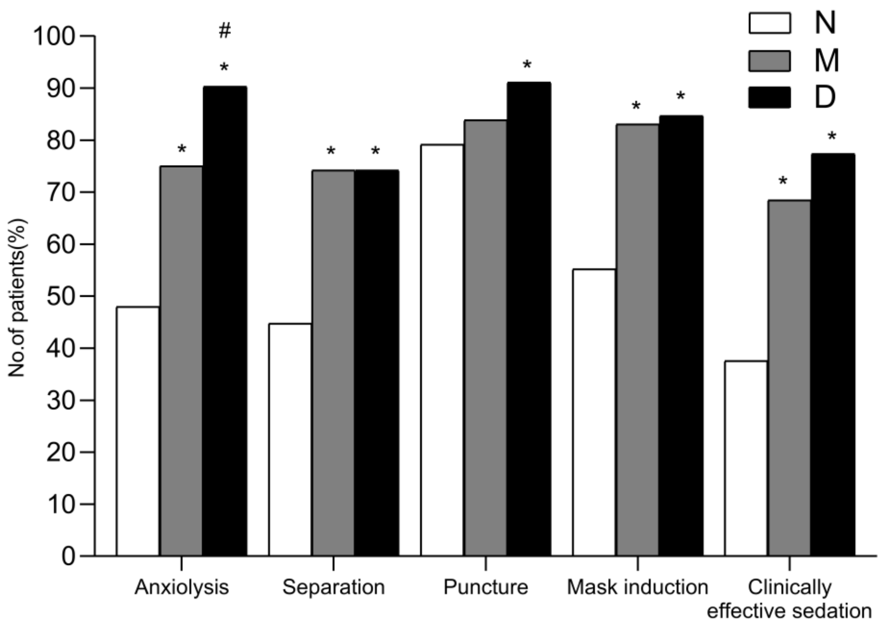

eFigure 2. Sedation Success Rate Among the Three Groups

Success rates for anxiolysis, separation, puncture, mask induction and clinically effective sedation before induction of anesthesia in normal saline, midazolam and dexmedetomidine groups (success=score of 3 or 4, clinically effective sedation=Total funk score ≥12. *$P<.017$, compared with Normal saline group；#$P<.017$, compared with Midazolam group.

© 2022 Shen F et al. *JAMA Network Open.*

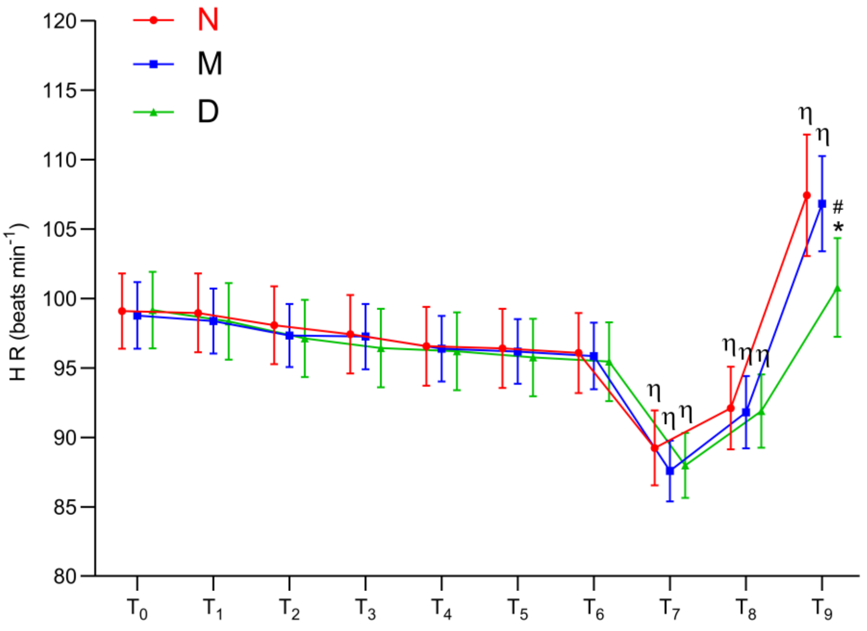

eFigure 3. Heart Rate Values at Different Times

Changes in heart rate were recorded at ten points [T_0, preadministration of premedication (baseline); T_1, 5 minutes after administration; T_2, 10 minutes after administration; T_3, 15 minutes after administration; T_4, 20 minutes after administration; T_5, 25 minutes after administration; T_6, 30 minutes after administration; T_7, after induction; T_8, after successful endotracheal tube insertion; T_9, after endotracheal tube removal]. HR, heart rate. $^*P < .017$, compared with Normal saline group; $^\# P < .017$, compared with Midazolam. $^\eta P < .001$, compared with T_0.

© 2022 Shen F et al. *JAMA Network Open.*
