# Supplementary material for: Effect of Intranasal Dexmedetomidine or Midazolam for Premedication on the Occurrence of Respiratory Adverse Events in Children Undergoing Tonsillectomy and Adenoidectomy: A Randomized Clinical Trial
Source: JAMA Netw Open. 2022 Aug 9;5(8):e2225473. doi: 10.1001/jamanetworkopen.2022.25473 (PMC9364121; doi:10.1001/jamanetworkopen.2022.25473)
Supplement: Supplement 3. — Data Sharing Statement [file jamanetwopen-e2225473-s003.pdf]

Data Sharing Statement

Shen. Effect of Intranasal Dexmedetomidine or Midazolam for Premedication on the Occurrence of Respiratory Adverse Events in Children Undergoing Tonsillectomy and Adenoidectomy. *JAMA Netw Open*. Published August 09, 2022. doi:10.1001/jamanetworkopen.2022.25473

Data
Data available: No
